# On the Origins of Homology Directed Repair in Mammalian Cells

**DOI:** 10.3390/ijms22073348

**Published:** 2021-03-25

**Authors:** Brett M. Sansbury, Eric B. Kmiec

**Affiliations:** Gene Editing Institute, Helen F. Graham Cancer Center & Research Institute, ChristianaCare, Newark, DE 19713, USA; Brett.Sansbury@ChristianaCare.org

**Keywords:** homology directed repair, gene editing, CRISPR-Cas, CRISPR

## Abstract

Over the course of the last five years, expectations surrounding our capacity to selectively modify the human genome have never been higher. The reduction to practice site-specific nucleases designed to cleave at a unique site within the DNA is now centerstage in the development of effective molecular therapies. Once viewed as being impossible, this technology now has great potential and, while cellular and molecular barriers persist to clinical implementations, there is little doubt that these barriers will be crossed, and human beings will soon be treated with gene editing tools. The most ambitious of these desires is the correction of genetic mutations resident within the human genome that are responsible for oncogenesis and a wide range of inherited diseases. The process by which gene editing activity could act to reverse these mutations to wild-type and restore normal protein function has been generally categorized as homology directed repair. This is a catch-all basket term that includes the insertion of short fragments of DNA, the replacement of long fragments of DNA, and the surgical exchange of single bases in the correction of point mutations. The foundation of homology directed repair lies in pioneering work that unravel the mystery surrounding genetic exchange using single-stranded DNA oligonucleotides as the sole gene editing agent. Single agent gene editing has provided guidance on how to build combinatorial approaches to human gene editing using the remarkable programmable nuclease complexes known as Clustered Regularly Interspaced Short Palindromic Repeats (CRISPR) and their closely associated (Cas) nucleases. In this manuscript, we outline the historical pathway that has helped evolve the current molecular toolbox being utilized for the genetic re-engineering of the human genome.

## 1. Introduction

Long before the advent of programmable nucleases, the field of gene editing was advancing methodically toward elucidating the molecular mechanism by which mammalian genes can be manipulated specifically by exogenously added reagents. In almost all cases, site-specific changes were executed by single-stranded DNA oligonucleotides (ssODNs) that penetrated the nuclear membrane and invaded the double helix providing the information to facilitate the desired nucleotide exchange. These early studies provided guidance toward understanding how genomic changes are executed [1,2]. As gene editing technologies advance, recent studies centered on the combinatorial action of ssODNs and programmable nucleases, such as CRISPR-Cas complexes, have continued along this line of investigation, elucidating our understanding of how eukaryotic genes can be genetically re-engineered. In this manuscript, we define the origins and the evolution of the gene editing reaction with a specific focus on homology directed repair.

## 2. Improving Efficiency by Modifying the Donor DNA

The gene editing strategy utilizing the simple addition of single-stranded DNA templates, known as single agent gene editing, takes place in a two-phase reaction, pairing and repairing [3]. At its most fundamental level, the oligonucleotide is designed to pair with its complementary region within the target gene apart from a central base purposely constructed to create a mismatch (pairing). The endogenous DNA repair systems recognize the artificial mismatch and direct the resolution of the mis-paired base (repairing). Thus, gene repair or nucleotide exchange is facilitated by the natural action of biological pathways inherent in the cell.

Seminal studies conducted in *E. coli* and Saccharomyces cerevisiae established the validity of synthetic DNA, ssODNs, as a donor template to direct site-specific base changes. Mandecki et al. [4] demonstrated permanent changes in plasmid DNA using oligonucleotides transformed into bacteria. Shortly thereafter, Sherman and colleagues published a series of important papers in which ssODNs were introduced into Saccharomyces cerevisiae with the goal of making inheritable changes in genes that determine the auxotrophic status of the organism [5]. Yammamoto et al. [6] targeted the CYC1 gene and described a distinct strand bias or polarity in the mutagenesis of gene editing reactions. Thus, foundational studies helped reveal the potential for using a simple strategy for making single base changes in chromosomal DNA.

The first transition to mammalian cells came from the work of Campbell et al. [7] who reported successful editing of an episomal target. The frequency with which gene repair took place was low, requiring a certain level of selection to identify cells that had undergone some degree of genetic alteration. Selection protocols were less effective in mammalian cells as the choice of reliable selection agents and protocols were limited, since they continue to be today. Thus, a more complete understanding of the rate limiting steps of gene editing was needed to provide information as to how the frequency of conversion in mammalian cells could be elevated.

**Figure 1:**
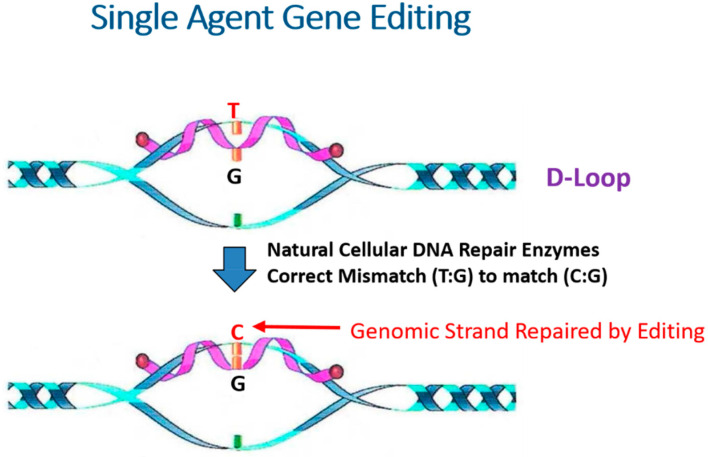
The D-Loop. A three stranded reaction intermediate is created when a single-stranded DNA molecule serving as the donor strand for homology directed repair interacts with this complementary strand within the helix. This step comprises the first phase of genetic engineering and DNA pairing. The dynamic binding of the single-stranded DNA with its complement creates the structure known as the D-loop. If a single nucleotide base is engineered into the donor strand so that it creates a mismatch with the nucleotide in the helix. Natural cellular DNA repair enzymes should then act to correct the mismatch and convert a mutant base to a normal base. This step comprises the second phase of gene editing known as DNA repairing.

The mechanism of action investigations into homologous pairing and strand exchange reactions, most often catalyzed by the RecA protein, revealed that the alignment of complementary stands into homologous register was a rate limiting step in the overall process [8,9]. Regardless of how the target site becomes available for pairing, the invasion of the single-stranded DNA into the duplex creates a key intermediate structure, the so-called D- loop, known as a three-stranded structure with a dynamic flux of unstable and stable base pairing in the region of complementarity (Figure 1). Once stabilized, the D-loop can be resolved in a variety of ways, leading to successful homologous exchange or disassembly of the pairing partners without genetic change. Importantly, the stability and half-life of the D-loop appears to be the governing factor in determining the outcome of homologous pairing. This remains true in CRISPR-directed gene editing since a series of molecular processes including replication, repair, and recombination take place within the interwoven complex of strands present at the invasion site.

Early efforts to increase stability of the three-stranded intermediate included the synthesis of triplex forming oligonucleotides (TFOs) and bifunctional oligonucleotides [10,11] and even DNA fragments tethered to TFOs [12]. Chemical and structural modifications showed some improvement including work by Ali-McNeer et al. [13] who tethered the TFO to a donor DNA strand. This complex was designed to repair the well-known F508 deletion mutation in Cystic Fibrosis. Significant correction of this mutation was achieved by creating a correction tool in which the targeting molecule was essentially constructed as a molecular clamp, positioning the active part to execute gene editing. In an alternative approach, RNA was incorporated directly into the single-stranded DNA oligonucleotide to create a chimeric oligonucleotide, which, by its very nature, elevated hybridization to the complementary DNA sequence at or near the target site and improved the targeting efficiency [9,14,15]. Then, in 2004, Drury et al. [16] provided confirmatory evidence that the D-loop is, in fact, the critical reaction intermediate in gene repair. Taken together, this work supported the concept that improving the stability of gene editing reaction intermediates could improve the efficiency with which overall gene editing or repair took place.

## 3. Enhancing the Frequency of Single Agent Gene Editing by Changing the Cellular Environment

One concern about extensive chemical modifications to the donor template was that they could lead to an immune response. Thus, while some workers continued to focus on the modification of the donor, other groups began to circle around the idea of modifying the cellular environment within which gene editing took place. By far, the most productive and successful strategy was the modulation of the cell cycle [1,17]. Whereas normal reactions using single-stranded DNA templates under standard cell growth conditions hovered between 0.3% and 1% at best, it was found that, if the donor templates were introduced into the cells during their transition through the S phase, the frequencies of gene editing increased three-fold to five-fold. Increasing the populations of cells in the S phase was achieved using cell synchronization with the population accumulating primarily at the G1/S border, followed by release into the S phase en masse. If transfection occurred after release, the DNA replication activity was slowed as the cells sensed an increased amount of DNA ends. This stalling engages the DNA damage response pathway and stimulates enzymatic activities required for gene editing [18]. The transitory slowdown of replication forks, in turn, enabled an even more proficient penetration of the chromatin structure by the single-stranded donor, once again enhancing target accessibility and improving gene editing frequency. The same sort of effect could also be obtained by incorporation of dideoxy compounds such as ddC, which would, in effect, slow the rate of DNA synthesis by reducing replication fork movement [19]. 

These discoveries have held true for elevating gene editing using CRISPR-Cas [20,21]. Lin et al. [22] demonstrated the same type of dependency using a Cas9 ribonucleoprotein complex in primary neonatal fibroblasts as well as human embryonic stem cells. Thus, the relationship established between gene editing and cell cycle progression for single agent activity has remained applicable to combinatorial gene editing using programmable nucleases.

## 4. Enhancing the Frequency of Single Agent Gene Editing Using Double-Strand DNA Breakage

Double-stranded breaks in chromosomes set in motion a cascade of events that lead to successful repair of the break, mutagenesis, or loss of cell viability. The repair of broken chromosomal DNA most often occurs by non-homologous end joining (NHEJ), which is a process where the broken ends of the DNA duplex are ligated back together to rescue the integrity of the chromosome and prevent mis-segregation at mitosis. Alternatively, the breaks in the chromosome can be restored by the process of homologous recombination, most often occurring in meiosis, and requiring a DNA repair template that bears some level of complementarity. The canonical pathway of NHEJ is operational through each phase of the cell cycle and is most likely the preferred pathway when chromosome integrity is in jeopardy. It requires far less energy [23,24,25]. Such activity serves to maintain accurate and balanced DNA content. A second type of non-homologous end joining is referred to as alternative NHEJ (alt-NHEJ), which is a process where double-strand breaks undergo a limited amount of resection and repair via micro-homology mediated end joining (MMEJ) [26,27]. While survival of the cell is the goal of such conjoining, NHEJ can be mutagenic, introducing insertions and/or deletions (indels) and breakpoint abnormalities. In contrast, homologous recombination maintains and even increases genetic diversity while ensuring genome integrity. Since double-strand DNA breaks can initiate genome rearrangement, and, with the emergence of programmable nucleases able to cleave at a specific site, the natural pathways of homologous recombination have become the foundation for the new age of genome engineering.

Workers studying single agent gene editing recognized that a double-strand break can also slow the progression of the cell cycle, impacting cells particularly transiting the S phase [28]. Thus, the generation of free ends ultimately consider recombinogenic can, in some cases, lead to the retardation of cells moving through S and G2 [18,19,29]. Under these conditions and in response to DNA damage, cell can once again become amenable to modification. Prior to the development of programmable nucleases, workers in the field used anticancer drugs such as VP16, bleomycin, or camptothecin to induce the double-strand breaks. The advantage of this approach is that a seamless delivery into the nucleus takes place since these drugs are small enough to penetrate the nuclear membrane without active transport. The downside is that multiple breaks occur at random within the chromosomal array, raising the possibility that unwanted and unwarranted mutagenesis could take place. Site-specific nuclease activity promoted by CRISPR-Cas systems have reduced the propensity for off-site cleavage because they can be designed to target and initiate a break site within the genome at the site designated for the change. This advance alone has been transformative for gene editing in mammalian cells. 

## 5. The Cellular Cost of Genome Engineering

**Figure 2:**
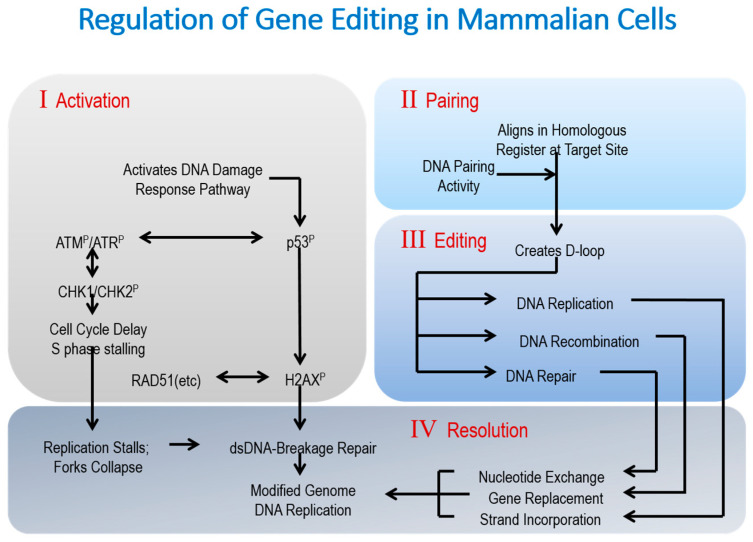
Regulation of gene editing in mammalian cells. In this diagram, traditional pairing and repairing (or resolution) phases of gene editing are expanded to include specific reactions that take place before, during, and after nucleotide exchange. Importantly, a whole series of metabolic events including DNA replication and DNA repair are active.

The delay in cell cycle progression has been termed reduced proliferation phenotype [30] (Figure 2). This phenotype emerges from observations in which the actual percentage of corrected cells within a population diminishes over time due, in large part, to the continual outgrowth of uncorrected cells. These cells are not adversely affected by the introduction of the single-stranded template, modified or not. The gross of the uncorrected cells reduces the percentage of cells that have been effectively edited through the process of simple dilution. The fact that only corrected cells exhibit this reduced proliferation phenotype leads to the conclusion that the chemical modification of single-stranded DNA template is not the primary cause for DNA damage activation of the slowdown in cell progression. It may be as simple as high levels of oligonucleotide, often required to direct effective nucleotide exchange in single agent gene editing, activate the DNA damage response, and subsequent reduction cell proliferation. Conversely, cells that have not received as much oligonucleotide remain unimpeded in terms of replication activity and, therefore, maintain a consistent level of proliferation.

## 6. Conclusions

The Nobel laureate Arthur Kornberg once said that it is a crime to waste clean thinking on dirty enzymes. His logic was based on the largely held tenet that, until we understand the activities of the fundamental components of a molecular reaction, using purified DNA templates and purified enzymes, we shall never truly appreciate how best to improve their collective activities. Early purveyors of gene editing followed this advice by utilizing purified donor DNA templates in the absence of other biomolecules. Single agent gene editing has been useful in mechanistic studies aimed at elucidating the regulatory circuitry surrounding gene editing in mammalian cells. Observations from studies in which single-stranded DNA oligonucleotides were the sole genetic tool have provided foundational information and important guidance to improve safe and efficacious genome modification. Chemical and structural modifications of the donor template improve stability. Some of the most important observations center on the concept that the cell must be viewed, in large part, as a living organism. Efforts to engineer the sophisticated tools to reach a specific outcome can have long-term and potentially mutagenic effects on the functionality and proliferation of the cells we aim to change. It will be interesting to see if we take advantage of what history has taught us.

## References

[1] Engstrom J.U., Kmiec E.B. (2008). DNA Replication, cell cycle progression and the targeted gene repair reaction. Cell Cycle.

[2] Parekh-Olmedo H., Ferrara L., Brachman E., Kmiec E.B. (2005). Gene therapy progress and prospects: Targeted gene repair. Gene Ther..

[3] Cole-Strauss A., Gamper H., Holloman W.K., Muñoz M., Cheng N., Kmiec E.B. (1999). Targeted gene repair directed by the chimeric RNA/DNA oligonucleotide in a mammalian cell-free extract. Nucleic Acids Res..

[4] Mandecki W. (1986). Oligonucleotide-directed double-strand break repair in plasmids of *Escherichia coli*: A method for site-specific mutagenesis. Proc. Natl. Acad. Sci. USA.

[5] Moerschell R.P., Tsunasawa S., Sherman F. (1988). Transformation of yeast with synthetic oligonucleotides. Proc. Natl. Acad. Sci. USA.

[6] Yamamoto T., Moerschell R.P., Wakem L.P., Komar-Panicucci S., Sherman F. (1992). Strand-specificity in the transformation of yeast with synthetic oligonucleotides. Genetics.

[7] Campbell C.R., Keown W., Lowe L., Kirschling D., Kucherlapati R. (1989). Homologous recombination involving small single-stranded oligonucleotides in human cells. New Biol..

[8] Symington L.S. (2014). End resection at double-strand breaks: Mechanism and regulation. Cold Spring Harb. Perspect. Biol..

[9] Kotani H., Kmiec E.B. (1994). A role for RNA synthesis in homologous pairing events. Mol. Cell. Biol..

[10] Culver K.W., Hsieh W.T., Huyen Y., Chen V., Liu J., Khripine Y., Khorlin A. (1999). Correction of chromosomal point mutations in human cells with bifunctional oligonucleotides. Nat. Biotechnol..

[11] Rogers F.A., Vasquez K.M., Egholm M., Glazer P.M. (2002). Site-directed recombination via bifunctional PNA-DNA conjugates. Proc. Natl. Acad. Sci. USA.

[12] Chan P.P., Lin M., Fawad Faruqi A., Powell J., Seidman M.M., Glazer P.M. (1999). Targeted correction of an episomal gene in mammalian cells by a short DNA fragment tethered to a triplex-forming oligonucleotide. J. Biol. Chem..

[13] McNeer N.A., Anandalingam K., Fields R.J., Caputo C., Kopic S., Gupta A., Quijano E., Polikoff L., Kong Y., Bahal R. (2015). Nanoparticles that deliver triplex-forming peptide nucleic acid molecules correct F508del CFTR in airway epithelium. Nat. Commun..

[14] Gamper H.B., Parekh H., Rice M.C., Bruner M., Youkey H., Kmiec E.B. (2000). The DNA strand of chimeric RNA/DNA oligonucleotides can direct gene repair/conversion activity in mammalian and plant cell-free extracts. Nucleic Acids Res..

[15] Gamper H. (2000). A plausible mechanism for gene correction by chimeric oligonucleotides. Biochemistry.

[16] Drury M.D., Kmiec E.B. (2004). Double displacement loops (double d-loops) are templates for oligonucleotide-directed mutagenesis and gene repair. Oligonucleotides.

[17] Aarts M., Te Riele H. (2011). Progress and prospects: Oligonucleotide-directed gene modification in mouse embryonic stem cells: A route to therapeutic application. Gene Ther..

[18] Olsen P.A., Randol M., Krauss S. (2005). Implications of cell cycle progression on functional sequence correction by short single-stranded DNA oligonucleotides. Gene Ther..

[19] EE Brachman E.K., Brachman E.E., Kmiec E.B. (2005). Gene repair in mammalian cells is stimulated by the elongation of S phase and transient stalling of replication forks. DNA Repair.

[20] Cong L., Ran F.A., Cox D., Lin S., Barretto R., Habib N., Hsu P.D., Wu X., Jiang W., Marraffini L.A. (2013). Multiplex Genome Engineering Using CRISPR/Cas Systems. Science.

[21] Bialk P., Rivera-Torres N., Strouse B., Kmiec E.B. (2015). Regulation of Gene Editing Activity Directed by Single-Stranded Oligonucleotides and CRISPR/Cas9 Systems. PLoS ONE.

[22] Schumann K., Lin S., Boyer E., Simeonov D.R., Subramaniam M., Gate R.E., Haliburton G.E., Ye C.J., Bluestone J.A., Doudna J.A. (2015). Generation of knock-in primary human T cells using Cas9 ribonucleoproteins. Proc. Natl. Acad. Sci. USA.

[23] Brandsma I., Gent D.C. (2012). Pathway choice in DNA double strand break repair: Observations of a balancing act. Genome Integr..

[24] Rodgers K., McVey M. (2016). Error-prone repair of DNA double-strand breaks. J. Cell. Physiol..

[25] Helleday T., Lo J., van Gent D.C., Engelward B.P. (2007). DNA double-strand break repair: From mechanistic understanding to cancer treatment. DNA Repair.

[26] Chang H.H.Y., Pannunzio N.R., Adachi N., Lieber M.R. (2017). Non-homologous DNA end joining and alternative pathways to double-strand break repair. Nat. Rev. Mol. Cell Biol..

[27] Wang H., Xu X. (2017). Microhomology-mediated end joining: New players join the team. Cell Biosci..

[28] Ferrara L., Engstrom J.U., Schwartz T., Parekh-Olmedo H., Kmiec E.B. (2007). Recovery of cell cycle delay following targeted gene repair by oligonucleotides. DNA Repair.

[29] Wu X.S., Xin L., Yin W.X., Shang X.Y., Lu L., Watt R.M., Cheah K.S.E., Huang J.D., Liu D.P., Liang C.C. (2005). Increased efficiency of oligonucleotide-mediated gene repair through slowing replication fork progression. Proc. Natl. Acad. Sci. USA.

[30] Engstrom J.U., Suzuki T., Kmiec E.B. (2009). Regulation of targeted gene repair by intrinsic cellular processes. 2BioEssays.

